# The protective role of prolyl oligopeptidase (POP) inhibition in acute lung injury induced by intestinal ischemia-reperfusion

**DOI:** 10.18632/oncotarget.28041

**Published:** 2021-08-17

**Authors:** Giovanna Casili, Sarah Adriana Scuderi, Marika Lanza, Alessia Filippone, Rossella Basilotta, Deborah Mannino, Michela Campolo, Emanuela Esposito, Irene Paterniti

**Affiliations:** ^1^Department of Chemical, Biological, Pharmaceutical and Environmental Sciences, University of Messina, Messina, Italy

**Keywords:** intestinal ischemia reperfusion (II/R), acute lung injury (ALI), prolyl oligopeptidase (POP), inflammation, angiogenesis

## Abstract

Intestinal ischemia-reperfusion (II/R) develops when the blood flow to the intestines decreases, followed by the reestablishment of the blood supply to the ischemic tissue, resulting in intestinal mucosal barrier dysfunction, with consequent severe local and systemic inflammation. Acute lung injury (ALI) represents the most serious complication after II/R. KYP-2047 is a selective inhibitor of prolyl oligopeptidase (POP), a serine protease involved in the release of pro-angiogenic and inflammatory molecules. The aim of the present study is to assess the effects of POP-inhibition mediated by KYP-2047 treatment in the pathophysiology of ALI following II/R. An *in vivo* model of II/R was performed and mice were subjected to KYP-2047 treatment (intraperitoneal, 1, 2.5 and 5 mg/kg). Histological analysis, Masson’s trichrome staining, immunohistochemical, immunofluorescence, biochemical and western blots analysis were performed on ileum and lung samples. KYP-2047 treatment ameliorated histological alteration in ileum and lung, reduced collagen amount and lowered inflammatory protein levels. Moreover, TGF-β1, eNOS, VEGF and CD34 positive staining has been modulated; also, a reduction in apoptosis expression was confirmed. This research revealed the strong anti-inflammatory potential of KYP-2047 associated to its modulatory role on angiogenesis and apoptosis, suggesting POP as a novel therapeutic target for ALI after II/R.

## INTRODUCTION

Ischemic process causes a lack of oxygen supply and nutrients and the following restoration of blood circulation, called reperfusion, results in oxidative tissue damage and invasion of inflammatory mediators to neighboring organs [1–3]. Intestinal ischemia-reperfusion (II/R) is a common route for many disorders, including enterocolitis, midgut volvulus, intussusception, intestinal obstruction, sepsis and hemodynamic shock. II/R damage takes over when the decrease of blood flow in the intestine is followed by the restoration of blood required to the ischemic area, resulting in severe local and systemic inflammation which spreads to nearby organs [4]. Acute lung injury (ALI) represents the most serious complication after intestinal I/R [5]. ALI is a medical condition characterized by widespread organ inflammation with an acute onset and, although several pathophysiologic mechanisms of ALI in II/R have been partially proposed, the basic concepts remain mostly vague [6].

II/R represents an classical example of critical illness, reporting the initiation and propagation of multiorgan failure; in this context, inflammation is an hallmark and various animal models and clinical data support the concept that excessive increase of proinflammatory cytokines is the main accountable in distant organ injury following II/R [7–9]. Interestingly, II/R provokes an important inflammatory response in nearby lung tissues, evidenced by neutrophilic infiltration, amplified myeloperoxidase levels and prominent vascular permeability in the lungs [6]. Moreover, the inflammatory cytokines increase outcomes in pulmonary damage, necrosis and apoptosis [3].

Reperfusion is responsible to magnifying the ischemic damage and provoke organ injuries and dysfunction through the overproduction of pro-inflammatory cytokines and the activation of angiogenesis process. Angiogenesis mechanism involves the sprouting of new blood vessels contributing to tissue repair, although the finality of the angiogenic response in acute ischemic stroke has not been fully elucidated; so, considering the importance of maintaining alveolar perfusion, the elucidation of microvascular angiogenetic mechanisms in lung, could be an interesting approach in damage [10].

The pathophysiology regarding lung diseases is complicated and proteolytic enzymes may be involved as possible biomarkers, among these an important role is done by prolyl endo or oligopeptidase (PREP or POP), a protease involved in angiogenesis process [11, 12]. Specifically, POP itself plays a role in supporting neutrophilic inflammation and this aspect involves POP to the pathology of various lung diseases [13]. Since the POP’s involvement in angiogenesis and inflammation has been highlighted [14], POP inhibitors have been developed and, between these, 4-phenyl-butanoyl-l-prolyl-2(S)-cyanopyrrolidine (KYP-2047) appears to be the most powerful and extensively studied both in *in vitro* and *in vivo* models of inflammatory diseases [12, 15, 16, 17]. Based on these findings, the aim of the present study was to assess the beneficial outcomes of POP-inhibition in lung disease induced by an experimental mouse model of intestinal ischemia performed by SAO shock-mediated injury.

## RESULTS

### Histological effects of POP-inhibition on lung damage induced by II/R

**Figure 1:**
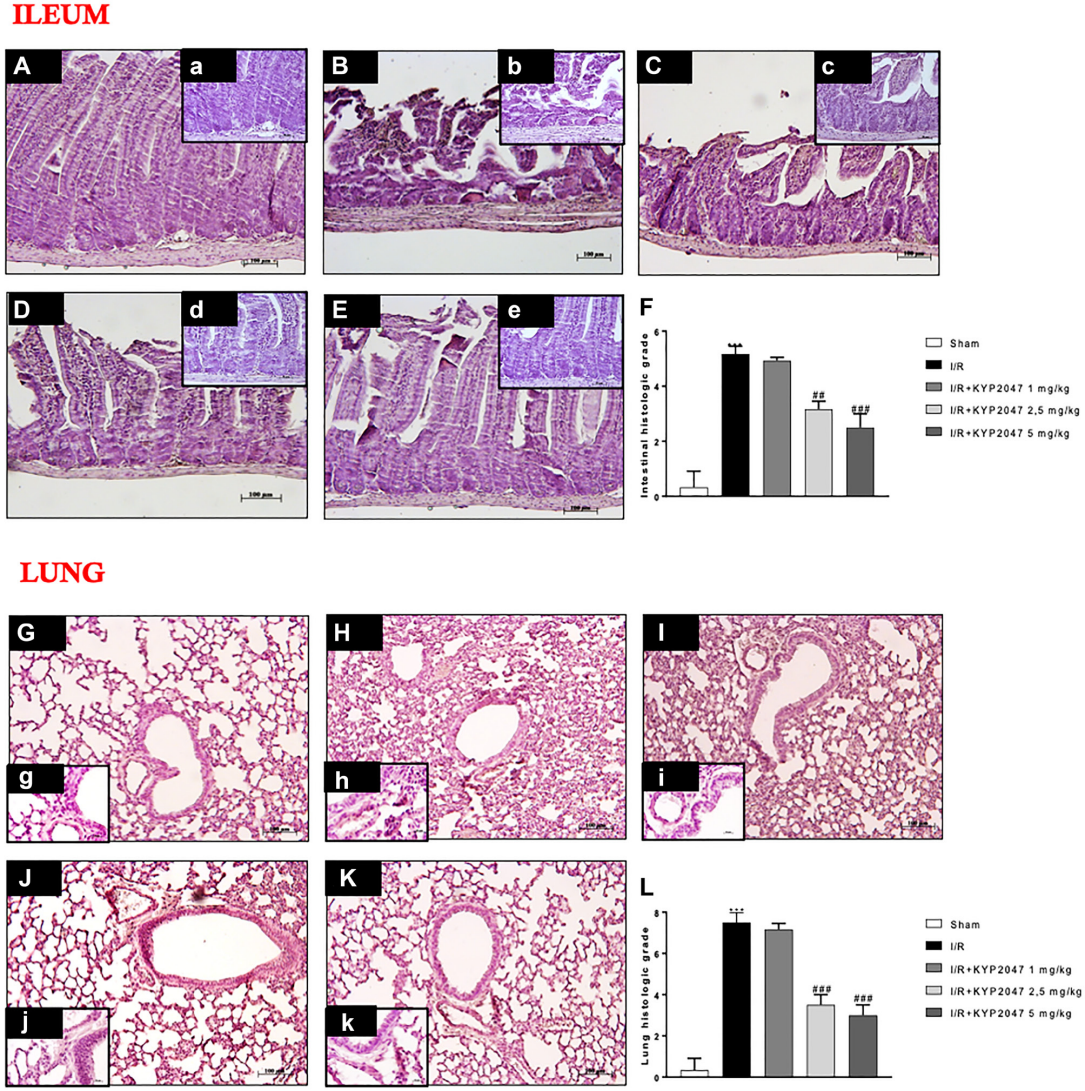
Role of KYP-2047 treatment on histological lung damage induced by II/R. (**A**, (a)) Histopathologic examination of ileum samples in sham group; (**B**, (b)) severe histological alteration with edema of intestinal villi in I/R group; (**C**, (c)) treatment with KYP-2047 1 mg/kg does not improve the histological picture; (**D**, (d), **E**, (e)) treatment with KYP-2047 at the doses of 2.5 and 5 mg/Kg restored the cytoarchitecture of ileum samples; (**F**) histological score. (**G**, (g)) Histopathologic examination of lung samples in sham group; (**H**, (h)) severe lung damage in I/R group; (**I**, (i)) POP-inhibition mediated by KYP-2047 treatment at the doses of 1 mg/kg and (**J**, (j), **K**, (k)) and at the doses of 2.5 and 5 mg/Kg, the only doses that significantly modulated lung histological impairment. (**L**) histological score. Magnification 10x, scale bar 100 μm (Figures A, B, C, D, E, G, H, J, K, L) and 20×, scale bar 50 μm (Figures a, b, c, d, e, g, h, j, k, l). Data represent the means of at least three independent experiments. One-way ANOVA followed by Bonferroni post-hoc. ^***^ *p* < 0.001 versus Sham; ^##^ *p* < 0.01 and ^###^ *p* < 0.001 versus II/R.

The histopathologic examination of ileum samples highlighted severe histological alteration characterized by edema in the intestinal villi (Figure 1B, (b) see histological score Figure 1F) compared to control group (Figure 1A, (a) see histological score Figure 1F). Treatment with KYP-2047, only at the doses of 2.5 and 5 mg/Kg, significantly reduced histological alteration, restoring the cytoarchitecture of the villi (Figure 1D, (d) and 1E, (e), see the histological score Figure 1F), unlike treatment at 1 mg/kg (Figure 1C, (c), see the histological score Figure 1F). To confirm that ALI is the most serious complication of intestinal I/R injury [18], the histological analysis was performed also on lung samples from II/R-injured mice, pointing out severe lung injury, as indicated by substantial alveolar edema, inflammatory cellular sequestration and hemorrhage (Figure 1H, (h), see the histological score Figure 1L), whereas the samples from control group exhibited normal lung histology (Figure 1G, (g), see the histological score Figure 1L). Interestingly, POP-inhibition mediated by KYP-2047 treatment, at the doses of 2.5 and 5 mg/Kg, significantly modulated lung histological impairment, enhancing these morphological changes (Figure 1J, (j) and 1K, (k), see the histological score Figure 1L), while the lowest dosage of 1 mg/Kg was unable to significantly preserve the lungs alteration from II/R damage (Figure 1I, (i), see the histological score Figure 1L).

### Role of KYP-2047 treatment in collagen content reduction on pulmonary damage induced by II/R

**Figure 2:**
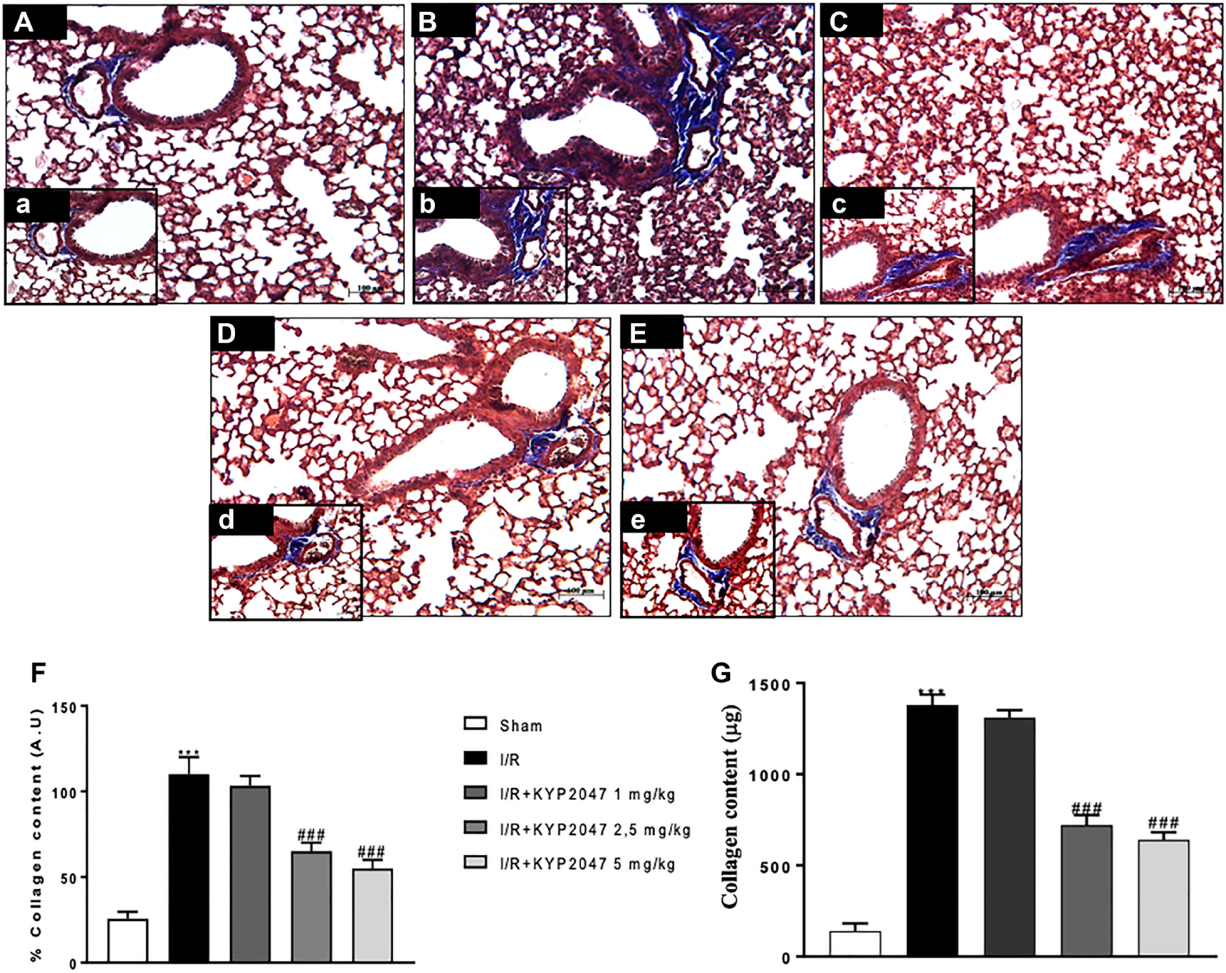
Role of KYP-2047 treatment on the collagen content. (**A**, (a)) lung samples from control group; (**B**, (b)) pulmonary fibrosis, assessed by Masson trichrome, stained in blue was greater in the lungs after II/R group; (**C**, (c)) treatment with KYP-2047 at 1 mg/kg did not reduced collagen depot, while, there was a significant reduced collagen depot, at the dose of 2.5 mg/Kg and 5 mg/Kg treatment (**D**, (d) and **E**, (e)); (**F**) % collagen content. These data were confirmed through the evaluation of lung collagen content (μg) (**G**). Magnification 10×, scale bar 100 μm (Figures A, B, C, D and E) and 20×, scale bar 50 μm (Figures a, b, c, d, and e). Data represent the means of at least three independent experiments. One-way ANOVA followed by Bonferroni post-hoc. ^***^ *p* < 0.001 versus Sham; ^###^ *p* < 0.001 versus II/R.

Pulmonary fibrosis stimulates alveolar filling with collagen-rich matrix, as common pathological response to lung damage; while many different etiologies provokes a fibrotic response in lung, II/R is able to significantly enhance the persistence and progression of fibrotic state [19, 20]. Thus, this study highlighted that the degree of fibrosis, evaluated by Masson trichrome staining, and demonstrated that fibrotic area (blue staining) was greater in the lungs subjected to II/R (Figure 2B, (b), see % collagen content Figure 2F) compared to lung samples from control group (Figure 2A, (a), see % collagen content Figure 2F). Treatment with KYP-2047, 5 minutes prior reperfusion, significantly reduced collagen depot, at the dose of 2.5 mg/Kg, almost similar to 5 mg/Kg treatment (Figure 2D, (d) and Figure 2E, (e), see % collagen content Figure 2F); instead, treatment of KYP-2047 at 1 mg/kg did not reduce collagen accumulation (Figure 2C, (c) see % collagen content Figure 2F). These data were confirmed through the evaluation of lung collagen content (μg) (Figure 2G), emphasizing the role of POP-inhibition to counteract collagen accumulation in lung samples after II/R (Figure 2G).

### Effects of KYP-2047-mediated POP inhibition on lung inflammation induced by II/R

II/R injury determinates a local intestinal and remote lung tissue pathology, described by a marked systemic inflammation [21]. Specifically, amplified activation of NF-κB has been observed associated to additional confirmation for a pro-inflammatory function for NF-κB in the lung resulting in neutrophilic infiltration and pulmonary oedema [22, 23]. Therefore, in this study, the activation of NF-κB pathway was investigated through Western Blot analysis.

**Figure 3:**
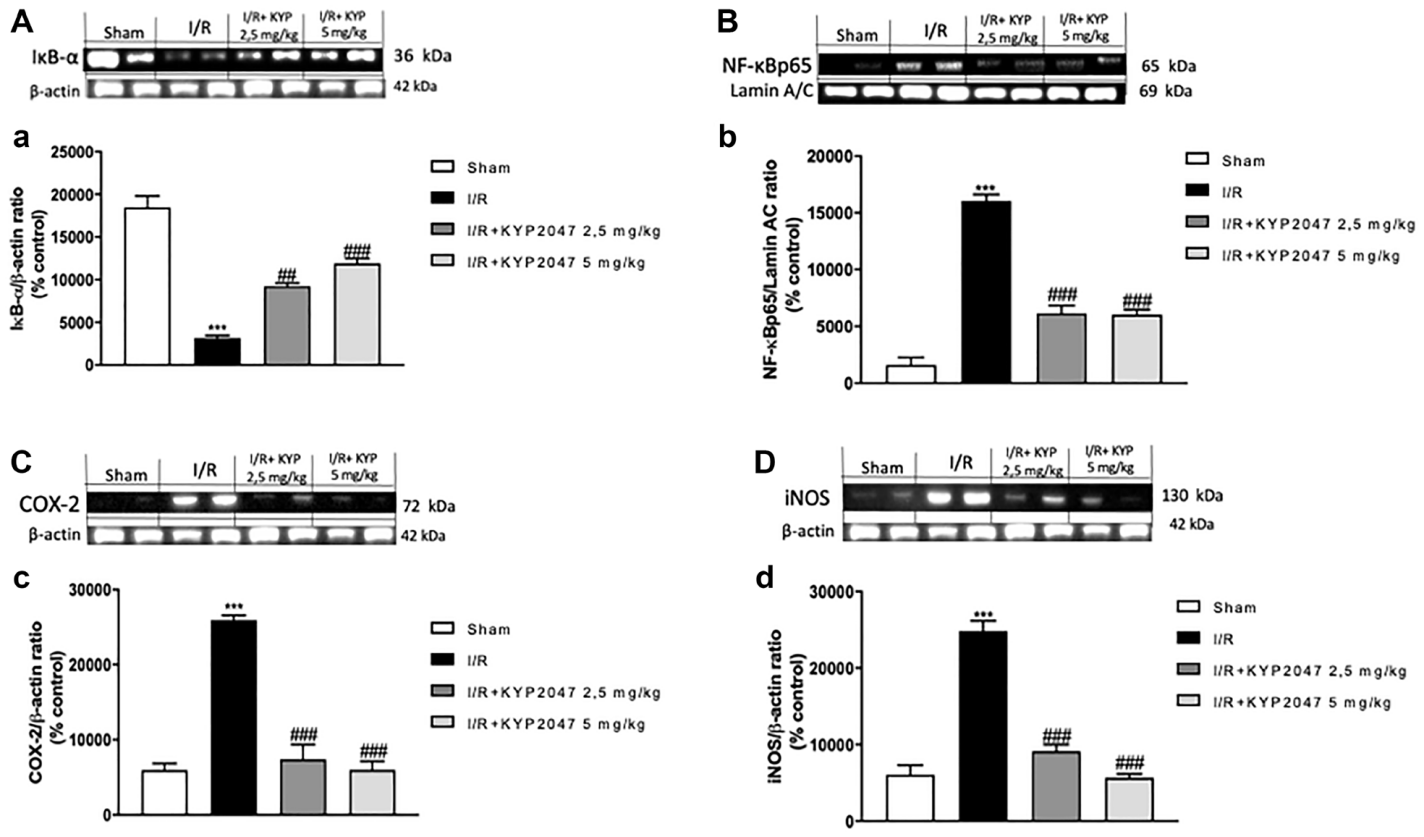
KYP-2047 treatment on lung inflammation. The protein levels of inflammatory markers were monitored by Western blotting in lung tissue. Basal levels of IκB-α were detected in lungs from sham mice (**A**, see densitometric analysis (a)), whereas the IκB-α expression were reduced in lung samples from damaged animals (A, see densitometric analysis (a)). KYP-2047 treatment, at both doses of 2.5 and 5 mg/Kg, was able to prevent the II/R-induced IκB-α cytosolic degradation (A, see densitometric analysis (a)). Similarly, nuclear NF-κB translocation was increased in lung samples from mice II/R-damaged compared to control group (**B**, see densitometric analysis (b)), while POP-inhibition through KYP2047 treatments, significantly reduced the nuclear translocation of p65 (B, see densitometric analysis (b)). Moreover, II/R subjected animals showed an increased expression of COX-2 and iNOS in lung samples compared to sham mice (respectively, **C**, see densitometric analysis (c) and **D**, see densitometric analysis (d); KYP-2047 treatment, at both doses of 2.5 and 5 mg/Kg, was able to reduce the expression of these inflammatory markers in a significant way (respectively, C, see densitometric analysis (c) and D, see densitometric analysis (d). Data represent the means of at least three independent experiments. One-way ANOVA followed by Bonferroni post-hoc. ^***^ *p* < 0.001 versus Sham; ^##^ *p* < 0.01 and ^###^ *p* < 0.001 versus II/R.

Basal levels of IκB-α were identified in lungs from sham mice (Figure 3A, see densitometric analysis (a)), whereas the IκB-α expression were reduced in lung samples of animals subjected to II/R (Figure 3A, see densitometric analysis (a)). KYP-2047 treatment, at both doses of 2.5 and 5 mg/Kg, was able to prevent the II/R-induced IκB-α cytosolic degradation (Figure 3A, see densitometric analysis (a)). Similarly, nuclear NF-κB translocation was increased in lung samples from mice II/R-damaged compared to control group (Figure 3B, see densitometric analysis (b)), while POP-inhibition through KYP2047 treatments, appreciably reduced the nuclear translocation of p65 (Figure 3B, see densitometric analysis (b)). Moreover, on the basis that iNOS and COX-2 enzymes represent important endogenous receptor targets for amplifying NF-κB responses in pathology [24], we also evaluated the expression of these enzymes on lung samples by Western Blot analysis. II/R subjected animals showed an increased expression of both COX-2 and iNOS in lung samples compared to sham mice (respectively, Figure 3C, see densitometric analysis (c) and Figure 3D, see densitometric analysis (d)); whereas KYP-2047 treatment, at both doses of 2.5 and 5 mg/Kg, was able to reduce the expression of these inflammatory markers in a significant way (respectively, Figure 3C, see densitometric analysis (c) and Figure 3D, see densitometric analysis (d)).

### Role of KYP-2047-mediated POP inhibition to modulate angiogenesis on lung after II/R injury

**Figure 4:**
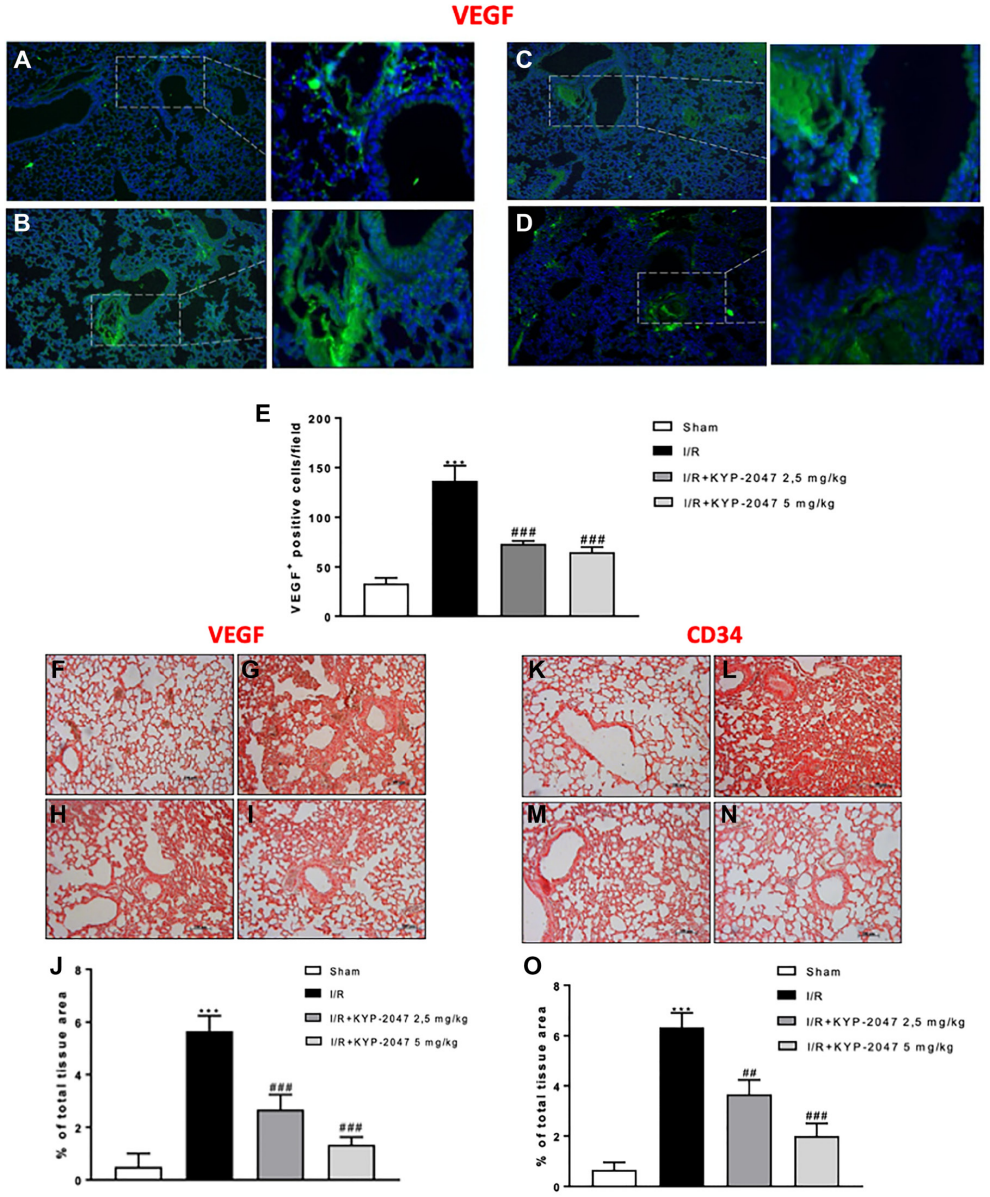
KYP-2047 treatment on angiogenesis. Immunofluorescence analysis on VEGF was performed, highlighting a lower VEGF positive staining in lung sections from control (**A**) compared to II/R group (**B**); KYP2047 treatment significantly reduced the VEGF positive staining in lung (**C**, **D**); (**E**) VEGF positive cells score and **H**, **I** see E and **J**). Moreover, the positive staining evaluated by immunohistochemistry analysis, Immunoistochemistry analysis on VEGF highlighted a reduced positive staining in control group (**F**) compared to II/R injury group (**G**); treatments with KYP-2047 (H, I); (J) % of total tissue area score for VEGF. The same data obtained for CD34 immunoistochemistry analysis; (**K**) control group, (**L**) II/R damaged group and (**M**, **N**) KYP-2047 treatment groups; (**O**) % of total tissue area score for CD34. Data represent the means of at least three independent experiments. One-way ANOVA followed by Bonferroni post-hoc. ^***^ *p* < 0.001 versus Sham; ^##^ *p* < 0.01 and ^###^ *p* < 0.001 versus II/R.

Inflammation arising from the ischemic process can induce lung angiogenesis [25]; also, the vascular VEGF protein is released by alveolar cell-like cell lines as reply to inflammatory stimuli potentially involved in lung injury, tending to increase in bronchoalveolar lavage fluid protein with substantial VEGF localization to lung epithelium [26]. To emphasize the *in vivo* modulatory action of KYP-2047 on angiogenesis in lung, an immunofluorescence and immunohistochemistry analysis on VEGF was performed. The study confirmed the increased VEGF positive staining in lung sections from II/R group (Figure 4B and 4G, see VEGF positive score Figure 4E and 4J) compared to control group (Figure 4A and 4F, see VEGF positive score Figure 4E and 4J; KYP2047 treatments significantly reduced the VEGF positive staining (Figure 4C, 4D and 4H, 4I see VEGF positive score Figure 4E and 4J). Moreover, previous studies on ischemia highlighted VEGF expression as key regulator of enhanced vascular and endothelial cell permeability, maintaining the characteristics of CD34+ cells, that are found in newly formed blood vessels under pathological conditions, connected with an augmented restoration of blood flow [27]. The positive staining evaluated by immunohistochemistry analysis, for CD34 in II/R injury group (Figure 4L, see VEGF positive score Figure 4O) was significantly lowered in lung sections from control group (Figure 4K, 4L, see VEGF positive score Figure 4O) and from KYP-2047 treated groups (Figure 4M, 4N, see VEGF positive score Figure 4O).

### The effects of KYP-2047-mediated POP inhibition to modulate lung injury following II/R damage through TGF-β1 and eNOS

**Figure 5:**
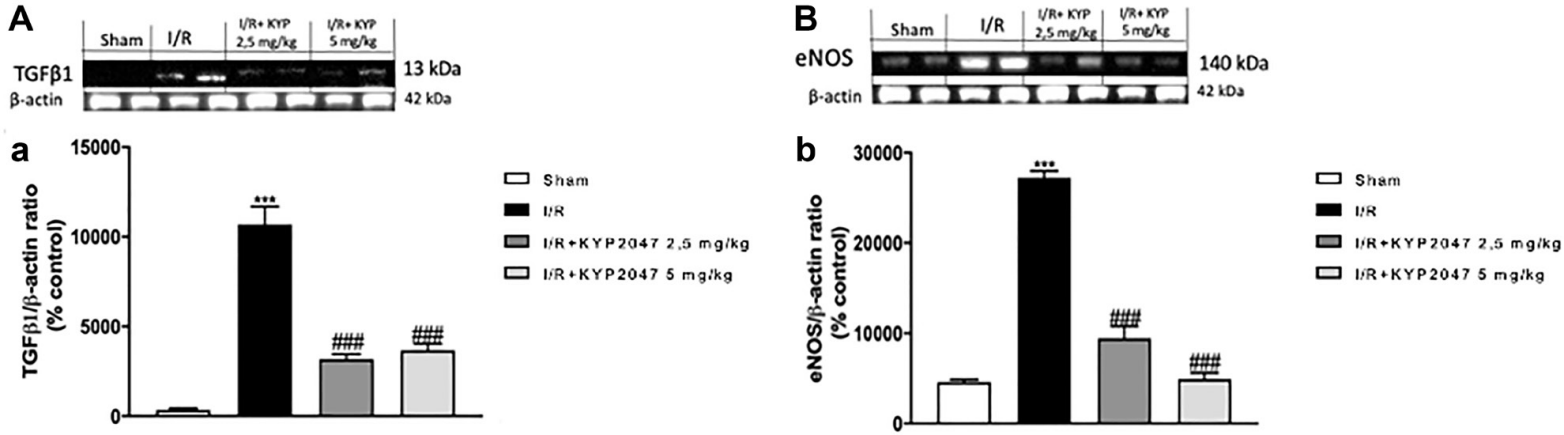
Role of KYP-2047 treatment on TGF-β1 and eNOS. Western Blot analysis for TGF-β1 and eNOS was performed, showing the upregulation of TGF-β1 in lung samples from II/R-injured mice compared to control group (**A**, see densitometric units score (a)). Interestingly, in lung samples from KYP-2047 treated group, at both doses of 2.5 and 5 mg/Kg, there was a significant reduction in TGF-β1 expression (A, see densitometric units score (a)). In the same time, Western Blot analysis for eNOS revealed a significant upregulation in lung samples from II/R-damaged animals compared to sham mice (**B**, see densitometric units score (b)); the POP inhibition, through treatment with KYP-2047, notably reduced the over expression of eNOS (B, see densitometric units score (b)). Data represent the means of at least three independent experiments. One-way ANOVA followed by Bonferroni post-hoc. ^***^ *p* < 0.001 versus Sham; ^###^ *p* < 0.001 versus II/R.

Endogenous TGF-β1 expression appears to be upregulated in the lung following II/R [28]; specifically, TGF-β1 is the most potent factor for the induction of myofibroblast differentiation and increased expression of TGF-β1 has been reported in fibrotic lungs [29]. In this study, through western blot analysis, the upregulation of TGF-β1 was confirmed in lung samples from II/R-injured mice compared to control group (Figure 5A, see densitometric units score (a)). Interestingly, lung samples from KYP-2047 treated group, at both doses of 2.5 and 5 mg/Kg, significantly reduced TGF-β1 expression (Figure 5A, see densitometric units score (a)), so promoting alveolar epithelial cell growth and repair. NO signal pathway could quickly increase TGF-β1, thereby stimulating pulmonary fibrosis [30] and inducing eNOS expression [31]. Thus, we detected by western blot analysis an upregulation of eNOS in lung samples from II/R-damaged animals compared to sham mice (Figure 5B, see densitometric units score (b)); whereas POP inhibition, through treatment with KYP-2047, notably reduced the over expression of eNOS (Figure 5B, see densitometric units score (b)).

### Role of POP inhibition on lung apoptosis following II/R damage

**Figure 6:**
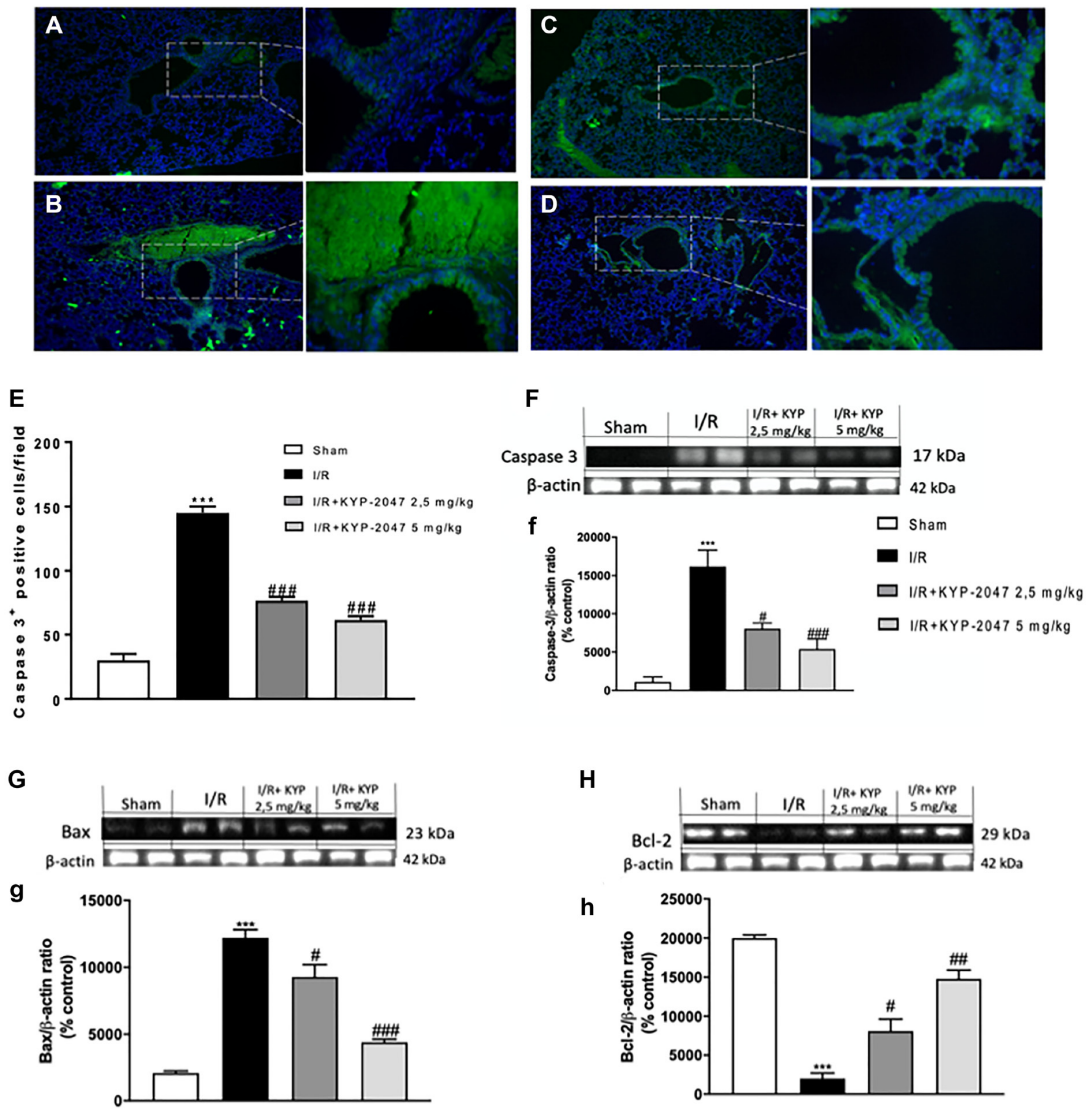
Role of KYP-2047 treatment on apoptosis. Immunofluorescence staining for Caspase-3 was performed to examine apoptosis in the lungs. Caspase-3 positive staining resulted lower in lung sections from sham group (**A**) compared to II/R-injured group (**B**); POP-inhibition mediated by KYP-2047 treatments, at both doses of 2.5 and 5 mg/Kg, significantly lowered the positive staining for Caspase-3 (**C** and **D**); (**E**) Caspase-3 positive score. These data were confirmed by Western Blot analysis (**F**, (f)). Moreover, through Western Blot analysis, a substantial Bax low expression, related to a notable increase in Bcl-2, was observed in lung samples from controls compared to II/R-injured mice, while treatment with KYP-2047 significantly reduced Bax protein expression, at the same time increasing the anti-apoptotic Bcl-2 protein levels (**G** and **H**, see densitometric units score (g) and (h)). Data represent the means of at least three independent experiments. One-way ANOVA followed by Bonferroni post-hoc. ^***^ *p* < 0.001 versus Sham; ^#^ *p* < 0.05, ^##^ *p* < 0.01 and ^###^ *p* < 0.001 versus II/R.

Among possible ALI’s mechanisms, cell apoptosis plays a predominant role; previous studies have described the ability of inflammation induces caspase activation in endothelial cells, which results in apoptosis induction and endothelial cell dysfunction [32]. In this study, to examine apoptosis immunofluorescence staining for Caspase-3 was performed. Interestingly, Caspase-3 positive staining resulted upregulated in lung sections from II/R-injured group (Figure 6B, see Caspase-3 positive score Figure 6E) compared to control (Figure 6A, see Caspase-3 positive score Figure 6E); POP-inhibition mediated by KYP-2047 treatment, at both doses of 2.5 and 5 mg/Kg, significantly lowered the positive staining for Caspase-3 (Figure 6C and 6D, see Caspase-3 positive score Figure 6E). These data were confirmed by western blot analysis for Caspase-3 protein expression (Figure 6F, (f)). It is known that small intestinal I/R induces apoptosis being the most sensitive organ to ischemic insult [33, 34] and, in particular, apoptosis as a mechanism of acute II/R provokes persistent lung damage, contributing to damage of both alveolar epithelial and endothelial barriers and causing an outflow of fluid and proteins into the alveolar airspace [35, 36]. For this reason, a Western Blot analysis was performed to highlight the role of Bax/Bcl-2 system in lung injury after SMA clumping damage. A substantial increase in Bax expression, related to a notable decrease in Bcl-2, was observed in lung samples from II/R-injured mice, compared to control group (Figure 6G and 6H, see densitometric units score (g) and (h)); treatment with KYP-2047 appreciably reduced Bax protein expression, at the same time increasing the anti-apoptotic Bcl-2 protein levels (Figure 6G and 6H, see densitometric units score (g) and (h)).

## DISCUSSION

Intestinal injury classically refers to a group of diseases characterized by a stoppage of the blood supply to various portions of small intestine, resulting in activation of ischemia process and secondary inflammatory changes; if untreated, this process causes life threatening intestinal necrosis. The keystones of modern treatment are an immediate diagnosis and an appropriate surgical intervention, needy for reduce the high mortality associated with this entity [37]. Intestinal injury is a clinical emergency that not only causes an arrest in oxygen supply to the metabolically active tissues but promotes, in the reperfusion phase, further cell damage [38]. Moreover, acute intestinal ischemia is an inevitable process in intestinal transplantation and surgical operations and acute lung damage represents a common cause of organ failure accompanying II/R [39, 40].

There are presently no evidence to pilot the evaluation or treatment of intestinal ischemia and its repercussions on neighboring organs and therapeutical guidelines have not been clearly defined [37]. Although numerous studies have searched the pathogenesis of II/R, little is known about alterations in organs other than heart, kidney and liver [36]. In fact, the role of II/R related-lung damage need to be better investigated and it is clinically relevant find solutions to prevent or mitigate ALI-induced by intestinal I/R.

Generally, the basic pathophysiology of lung diseases is complex and proteolytic enzymes may be intricate or could serve as potential biomarkers; in this context, POP emerges with a highly expression on inflammatory cells in different pulmonary pathologies [11]. Based on these evidences, the aim of this research was to evaluate the protective effect of KYP-2047, as POP inhibitor, to counteract inflammation, angiogenesis and apoptotic mechanisms involved in the pathophysiology of lung injury provoked by II/R damage on *in vivo* mouse model of SAO.

Intestinal ischemia provokes disruption of intestinal barrier, permitting bacteria translocation into the blood circulation, consequently exposing to the risk of a systemic inflammatory response and lung injury. II/R injury causes focal loss of surface epithelium and submucosal infarction characterized by loss of variable amounts of lamina propria, followed by severe remote lung tissue alterations, associated to marked neutrophil influx and edema formation [41, 42]. Interestingly, in this study was confirmed that treatment with KYP-2047, inhibiting POP activity, is able to restore the histological alterations caused by II/R both on small intestine and in lungs.

ALI starts from either a direct or an indirect alterations of the pulmonary epithelium and endothelium that causes edema, atelectasis and fibrosis [43]. Lung fibrosis is characterized by fibroblast accumulation that synthesize and deposit collagen and other extracellular matrix components [44]. In this study we observed that the treatment with KYP-2047 significantly reduced collagen distribution and content in the lung, highlighting a real protection of POP inhibition from fibrosis-related lung injury.

Although the pathophysiology of ALI after II/R remains intricate, it is extensively approved that inflammatory response is the primary mechanisms leading to pulmonary disease [45, 46]. Specifically, extensive evidence suggests that activation of NF-κB is a key factor in the regulation of inflammation and angiogenesis, influencing the lung response to II/R [23]. In this study, we proved the capacity of KYP-2047 treatment to counteract inflammation through a downregulation of NF-κB-pathway, action empowered from a reduction in inflammatory enzymes like iNOS and COX-2; in fact, in the current study, we found that POP inhibition, through KYP-2047 treatment, markedly inhibited the augmented NO levels induced by intestinal I/R injury, so highlighting the role of POP inhibitor to decrease inflammation in lung after II/R.

Inflammation process, after ischemia, is an important trigger for ischemia-induced angiogenesis, as inflammatory cells can release various angiogenic markers, including VEGF [47]; it is a notable chemokine that plays critical roles in angiogenesis and vasculogenesis, mediating tissue restoration in cases of ischemic injury [48]. The VEGF appearance is connected to the expression of the CD34+ antigen, detected in newly blood vessels under pathological conditions associated with a faster restoration of blood flow [49]. In this study, we demonstrated a significant reduction of VEGF and CD34 positive staining on lungs from KYP-2047 -treated groups, compared to the samples from II/R damaged group.

Moreover, besides VEGF, also TGF-β1 is overexpressed in patients after ischemic stroke, being central to the processes of angiogenesis, tissue inflammation and fibrosis [50]; furthermore, following ALI, TGF-β1 has been most carefully evaluated during the late phases of tissue repair, where it plays a critical role in the progress of lung fibrosis, increasing the permeability of endothelial monolayers [51]. In this study, we confirmed that KYP-2047 treatment significantly reduced TGF-β1 protein levels in lung injury following II/R. Many forms of vascular disease are characterized by increased TGF-β1 expression and endothelial dysfunction [31] with increased expression of eNOS; although the precise mechanism by which it does so is unclear probably, transcription factors activated by TGF-β1, mediates TGF-β1 induction of eNOS in endothelial cells [52]. In this study, we confirmed an increased expression of eNOS, related to TGF-β1, in II/R-damaged group, closely associated with a significative decrement in KYP-2047 treated groups.

There is agreement on the central role of NO in programmed cell death and the relevance of apoptosis following ischemia and reperfusion has been demonstrated [53, 54], underlying that protracted periods of ischemia are linked to an increased necrotic process, whereas, unexpectedly, reperfusion leads to an enhancement in apoptosis, restoring energy necessary for the apoptosis completion [55, 56]. We proved this apoptosis modulation also in lung following II/R, highlighting that KYP-2047 treatment acting through the activation of caspase enzymes and thus reducing apoptosis, may enhance preservation of the lung after II/R injury.

## MATERIALS AND METHODS

### Animals

CD1 male mice (25–30 g, 8–9 weeks Harlan, Italy) were kept in an organized environment with standard rodent chow and water. Animals were housed in stainless steel cages into a room at 22 ± 1°C with a 12-h light, 12-h dark cycle. The study was permitted by the University of Messina Review Board for the care of animals. All animal experiments were performed following the regulations in Italy (D.M. 116192), Europe (O.J. of E.C. L 358/1 12/18/1986), USA (Animal Welfare Assurance No A5594-01, Department of Health and Human Services, USA).

### II/R induction

The induction of splanchnic artery occlusion (SAO) was performed as previously described [57]. Briefly, a midline laparotomy was performed, identifying the superior mesenteric artery (SMA) and occluding below the celiac trunk with an arterial microclamp for 30 minutes. After this time, the clamp was removed, 0.5 mL of sterile saline at 37°C was injected into the peritoneal cavity, reperfusing the animals for 1 hour. Sham-operated mice experienced identical surgical interventions and time courses without SMA clamping. Intestinal ischemia was confirmed by whiteness of ileum. After the reperfusion period, animals were killed, ileum samples were collected to prove that reperfusion ischemia has occurred; also, lung samples were collected and processed for histological, immunohistochemical, immunofluorescence, biochemical and western blots analysis.

### Experimental groups

Mice were in random allocated in different groups:

1. Sham + vehicle group: mice were subjected to surgical procedures except for SAO shock and were kept under anesthesia for the time of the experiment (*n* = 10) plus administration of vehicle (saline).
2. Intestinal I/R + vehicle group: mice (*n* = 10) were subjected to intestinal ischemia by SAO (30 min), followed by reperfusion (1 h) plus administration of vehicle (saline).
3. Intestinal I/R + KYP-2047 (1 mg/Kg): mice were subjected to surgical procedures, described as above, and KYP-2047 was administered intraperitoneally (1 mg/kg) 5 min prior to reperfusion (*n* = 10).
4. Intestinal I/R + KYP-2047 (2.5 mg/Kg): mice were subjected to surgical procedures, described as above, and KYP-2047 was administered intraperitoneally (2.5 mg/kg) 5 min prior to reperfusion (*n* = 10).
5. Intestinal I/R + KYP-2047 (5 mg/Kg): mice were subjected to surgical procedures, described as above, and KYP-2047 was administered intraperitoneally (5 mg/kg) 5 min prior to reperfusion (*n* = 10).

KYP-2047 was dissolved in saline (0.001% DMSO) and administered according to the bibliography. The doses of KYP-2047 (1, 2.5 and 5 mg/kg) used for the experiment were based on literature and on previous *in vivo* study [12, 58].

### Histological analysis

Ileum and lung samples were collected and analyzed for histopathological examination, as previously described. Briefly, lungs were before fixed in 10% (w/v) PBS-buffered formalin and then 7-μm sections were prepared from paraffin-fixed tissues to perform H&E staining. The small intestine and lung injuries were graded in accordance with previously described scoring systems [42, 59]. The sections were evaluated by computer-assisted color image investigation (Leica QWin V3, Cambridge, UK). The histological results were showed 10× and 20× (100 and 50 μm of the Bar scale). All histopathological evaluations were executed in a blinded fashion by a pathologist who was unaware of the study details.

### Masson’s trichrome staining

To evaluate the degree of fibrosis, tissue sections from lungs were stained with Masson trichrome according to the manufacturer’s protocol (Bio-Optica, Milan, Italy), as previously described [60].

### Quantification of lung collagen

The quantification of lung collagen was performed as previously described [19]. Quickly, the pulmonary vasculature was perfused with sterile saline and left lungs were excised at the hilum, homogenized and then incubated in 65% trichloroacetic acid (Fisher Scientific, Pittsburgh, PA) on ice. The denatured tissues were incubated with 1.4% chloramine T (500 ml; Sigma- Aldrich, St Louis, MO) in 10% isopropanol and 0.5 M sodium acetate for 20 min at room temperature; 1 M 4-(dimethylamino)-benzaldehyde (500 ml; Sigma-Aldrich) was added and the suspension was incubated for 15 min at 65°C. Absorbance at 540 nm was measured in triplicate for each sample.

### Western blot analysis for NF-κB, IκB-α, Cyclooxygenase 2 (COX-2), Inducible nitric oxide synthase (iNOS), endothelial nitric oxide synthase (eNOS), Bax, Bcl-2, Caspase-3 and Transforming Growth Factor β (TGF-β1)

Total cytosolic and nuclear extracts were prepared, as previously described [61] on lung samples. The following primary antibodies were used: anti- NF-κB (Santa Cruz Biotechnology, 1:500 #sc8008, D.B.A, Milan, Italy), anti- IκB-α (Santa Cruz Biotechnology, 1:500 #sc1643, D.B.A, Milan, Italy),

anti-COX2 (Santa Cruz Biotechnology, 1:500 #sc376861, D.B.A, Milan, Italy), anti-iNOS (Abcam; 1:1000 #ab15323), anti-eNOS (Abcam [M221]; 1:1000 #ab76198, D.B.A, Milan, Italy), anti-BAX (Santa Cruz Biotechnology; 1:500 #sc7480), anti-Bcl2 (Santa Cruz Biotechnology; 1:500 #sc7382) and anti-Caspase-3 (Santa Cruz Biotechnology; 1:500 #sc7272) in 1× phosphate-buffer saline (Biogenerica srl, Catania, Italy), 5% w/v non-fat dried milk, 0.1% Tween-20 at 4°C overnight. Membranes were incubated with peroxidase-conjugated bovine anti-mouse IgG secondary antibody (Jackson ImmunoResearch, West Grove, PA, USA; 1:2000) for 1 h at room temperature. Anti-β-actin (Santa Cruz Biotechnology; 1:1000 #sc47778) and anti-Lamin A/C (Santa Cruz Biotechnology; 1:1000 #sc376248) antibodies were used as controls. Protein expression was detected by chemiluminescence (ECL) system (Thermo, Waltham, MA, USA), visualized with the ChemiDoc XRS (Bio-Rad, Hercules, CA, USA) and analyzed by using Image Lab 3.0 software (Bio-Rad, Hercules, CA, USA) and Image J software 1.53 version. The value of densitometric fold-changes was evaluated in protein amounts as ratio measure of bands represented in the blots (% control).

### Immunohistochemical analysis

Immunohistochemistry (IHC) staining for vascular endothelial growth factor (VEGF) and CD34 was measured in the lung tissues as previously described [62]. Briefly, lung sections (7 μm) were processed and incubated overnight with anti-VEGF polyclonal antibody (Santa Cruz Biotechnology; 1:500 #sc7269 in PBS, v/v, MA, USA) and anti-Caspase-3 (Santa Cruz Biotechnology; 1:500 #sc7272 in PBS, v/v, MA, USA). To verify antibody-binding specificity, control slices were incubated with only primary antibody or secondary antibody, neither of which gave positive staining. Images were collected using a Zeiss microscope and Axio Vision software. For graphic display of densitometric analyses, the % of positive staining (brown staining) was measured by computer-assisted color image analysis (Leica QWin V3, UK). The percentage area of immunoreactivity (determined by the number of positive pixels) was expressed as % of total tissue area (red staining) within five random fields at ×40 magnification.

### Immunofluorescence analysis

Detection of VEGF and Caspase-3 was carried out as previous described [63]. Briefly, lung section were incubated with mouse monoclonal anti-VEGF (1:100, Santa Cruz Biotechnology, Santa Cruz, CA, USA) or mouse monoclonal anti-caspase-3 (1:200, Santa Cruz Biotechnology, Santa Cruz, CA, USA). Sections were observed at 40× magnifications by a Leica DM2000 microscope (Leica, Milan, Italy). Examining the most luminously labeled pixels and using settings that allowed clear visualization of structural details, while keeping the maximum pixel intensities close to 200, established contrast and brightness. The same settings were used for all images obtained from the other samples that had been processed in parallel. Digital images were cropped and figure montages ready using Adobe Photoshop 7.0 (Adobe Systems; Palo Alto, California, United States).

### Materials

KYP-2047 (Sigma, cat#SML0208, Lot#032M4606V) was purchased by Sigma-Aldrich (Milan, Italy). All other compounds were obtained from Sigma-Aldrich (St. Louis, MO, USA). Each stock solution was prepared in non-pyrogenic saline (0.9% NaCl, Baxter, Milan, Italy).

### Statistical analysis

All values are indicated as mean ± standard error of the mean (SEM) of N observations. N represents the number of animals engaged. The experiment is descriptive of as a minimum of three experiments performed on different days on tissue sections collected from all animals in each experimental group. Data were analyzed with GraphPad 5 software, by one-way ANOVA followed by a Bonferroni post-hoc test for multiple comparisons. A *p*-value of less than 0.05 was considered significant.

## CONCLUSIONS

In conclusion, POP protein is connected to the pathogenesis of II/R and POP-inhibition mediated by KYP-2047 treatment has a protective role in lung injury induced by II/R. Although the specified molecular mechanisms of POP-inhibition remain to be clarified, these data recommend a convincing anti-inflammatory potential of KYP-2047 associated to its modulatory role on angiogenesis and apoptosis, that contribute to modulate intestinal/reperfusion damage and reduce lung injury resulting from II/R, suggesting POP as a novel therapeutic target for ALI after II/R.

## Abbreviations

POP: Prolyl oligopeptidase
PREP: Prolyl endopeptidase
II/R: Intestinal ischemia reperfusion
ALI: Acute lung injury
KYP-2047: 4-phenyl-butanoyl-l-prolyl-2(S)-cyanopyrrolidine
SAO: Splanchnic artery occlusion
SMA: Superior mesenteric artery
COX-2: Cyclooxygenase 2
iNOS: Inducible nitric oxide synthase
eNOS: Endothelial nitric oxide synthase
TGF-β1: Transforming Growth Factor β

## References

[1] Oldenburg WA, Lau LL, Rodenberg TJ, Edmonds HJ, Burger CD. Acute mesenteric ischemia: a clinical review. Arch Intern Med. 2004; 164:1054–62. 10.1001/archinte.164.10.1054. 15159262

[2] Breithaupt-Faloppa AC, Vitoretti LB, Cavriani G, Lino-dos-Santos-Franco A, Sudo-Hayashi LS, Oliveira-Filho RM, Vargaftig BB, Tavares-de-Lima W. Intestinal lymph-borne factors induce lung release of inflammatory mediators and expression of adhesion molecules after an intestinal ischemic insult. J Surg Res. 2012; 176:195–201. 10.1016/j.jss.2011.06.074. 21872880

[3] Ng CS, Wan S, Arifi AA, Yim AP. Inflammatory response to pulmonary ischemia-reperfusion injury. Surg Today. 2006; 36:205–14. 10.1007/s00595-005-3124-2. 16493527

[4] Pierro A, Eaton S. Intestinal ischemia reperfusion injury and multisystem organ failure. Semin Pediatr Surg. 2004; 13:11–17. 10.1053/j.sempedsurg.2003.09.003. 14765366

[5] Thais Fantozzi E, Rodrigues-Garbin S, Yamamoto Ricardo-da-Silva F, Oliveira-Filho RM, Spina D, Tavares-de-Lima W, Riffo-Vasquez Y. Acute lung injury induced by intestinal ischemia and reperfusion is altered in obese female mice. Pulm Pharmacol Ther. 2018; 49:54–59. 10.1016/j.pupt.2018.01.005. 29337267

[6] Ma Y, Zabell T, Creasy A, Yang X, Chatterjee V, Villalba N, Kistler EB, Wu MH, Yuan SY. Gut Ischemia Reperfusion Injury Induces Lung Inflammation via Mesenteric Lymph-Mediated Neutrophil Activation. Front Immunol. 2020; 11:586685. 10.3389/fimmu.2020.586685. 33042165 PMC7517702

[7] Souza DG, Cassali GD, Poole S, Teixeira MM. Effects of inhibition of PDE4 and TNF-alpha on local and remote injuries following ischaemia and reperfusion injury. Br J Pharmacol. 2001; 134:985–94. 10.1038/sj.bjp.0704336. 11682446 PMC1573029

[8] Souza DG, Cara DC, Cassali GD, Coutinho SF, Silveira MR, Andrade SP, Poole SP, Teixeira MM. Effects of the PAF receptor antagonist UK74505 on local and remote reperfusion injuries following ischaemia of the superior mesenteric artery in the rat. Br J Pharmacol. 2000; 131:1800–08. 10.1038/sj.bjp.0703756. 11139461 PMC1572514

[9] Sun Z, Wang X, Lasson A, Böjesson A, Annborn M, Andersson R. Effects of inhibition of PAF, ICAM-1 and PECAM-1 on gut barrier failure caused by intestinal ischemia and reperfusion. Scand J Gastroenterol. 2001; 36:55–65. 10.1080/00365520150218066. 11218240

[10] Arenillas JF, Sobrino T, Castillo J, Dávalos A. The role of angiogenesis in damage and recovery from ischemic stroke. Curr Treat Options Cardiovasc Med. 2007; 9:205–12. 10.1007/s11936-007-0014-5. 17601384

[11] Vliegen G, Raju TK, Adriaensen D, Lambeir AM, De Meester I. The expression of proline-specific enzymes in the human lung. Ann Transl Med. 2017; 5:130. 10.21037/atm.2017.03.36. 28462210 PMC5395489

[12] Casili G, Lanza M, Scuderi SA, Messina S, Paterniti I, Campolo M, Esposito E. The Inhibition of Prolyl Oligopeptidase as New Target to Counteract Chronic Venous Insufficiency: Findings in a Mouse Model. Biomedicines. 2020; 8:604. 10.3390/biomedicines8120604. 33322134 PMC7764674

[13] O’Reilly PJ, Hardison MT, Jackson PL, Xu X, Snelgrove RJ, Gaggar A, Galin FS, Blalock JE. Neutrophils contain prolyl endopeptidase and generate the chemotactic peptide, PGP, from collagen. J Neuroimmunol. 2009; 217:51–54. 10.1016/j.jneuroim.2009.09.020. 19875179 PMC2787998

[14] Myöhänen TT, Tenorio-Laranga J, Jokinen B, Vázquez-Sánchez R, Moreno-Baylach MJ, García-Horsman JA, Männistö PT. Prolyl oligopeptidase induces angiogenesis both *in vitro* and *in vivo* in a novel regulatory manner. Br J Pharmacol. 2011; 163:1666–78. 10.1111/j.1476-5381.2010.01146.x. 21133893 PMC3166694

[15] Rostami J, Jäntti M, Cui H, Rinne MK, Kukkonen JP, Falk A, Erlandsson A, Myöhänen T. Prolyl oligopeptidase inhibition by KYP-2407 increases alpha-synuclein fibril degradation in neuron-like cells. Biomed Pharmacother. 2020; 131:110788. 10.1016/j.biopha.2020.110788. 33152946

[16] Natunen TA, Gynther M, Rostalski H, Jaako K, Jalkanen AJ. Extracellular prolyl oligopeptidase derived from activated microglia is a potential neuroprotection target. Basic Clin Pharmacol Toxicol. 2019; 124:40–49. 10.1111/bcpt.13094. 29998529

[17] Jalkanen AJ, Leikas JV, Forsberg MM. KYP-2047 penetrates mouse brain and effectively inhibits mouse prolyl oligopeptidase. Basic Clin Pharmacol Toxicol. 2014; 114:460–63. 10.1111/bcpt.12184. 24350801

[18] Thomas S, Karnik S, Balasubramanian KA. Surgical manipulation of the small intestine and its effect on the lung. J Surg Res. 2002; 106:145–56. 10.1006/jsre.2002.6388. 12127820

[19] Chai D, Zhang L, Xi S, Cheng Y, Jiang H, Hu R. Nrf2 Activation Induced by Sirt1 Ameliorates Acute Lung Injury After Intestinal Ischemia/Reperfusion Through NOX4-Mediated Gene Regulation. Cell Physiol Biochem. 2018; 46:781–92. 10.1159/000488736. 29621765

[20] McKleroy W, Lee TH, Atabai K. Always cleave up your mess: targeting collagen degradation to treat tissue fibrosis. Am J Physiol Lung Cell Mol Physiol. 2013; 304:L709–21. 10.1152/ajplung.00418.2012. 23564511 PMC3680761

[21] Gifford AH, Matsuoka M, Ghoda LY, Homer RJ, Enelow RI. Chronic inflammation and lung fibrosis: pleotropic syndromes but limited distinct phenotypes. Mucosal Immunol. 2012; 5:480–84. 10.1038/mi.2012.68. 22806097

[22] Cheng DS, Han W, Chen SM, Sherrill TP, Chont M, Park GY, Sheller JR, Polosukhin VV, Christman JW, Yull FE, Blackwell TS. Airway epithelium controls lung inflammation and injury through the NF-kappa B pathway. J Immunol. 2007; 178:6504–13. 10.4049/jimmunol.178.10.6504. 17475880

[23] Alvira CM. Nuclear factor-kappa-B signaling in lung development and disease: one pathway, numerous functions. Birth Defects Res A Clin Mol Teratol. 2014; 100:202–16. 10.1002/bdra.23233. 24639404 PMC4158903

[24] Salvemini D, Kim SF, Mollace V. Reciprocal regulation of the nitric oxide and cyclooxygenase pathway in pathophysiology: relevance and clinical implications. Am J Physiol Regul Integr Comp Physiol. 2013; 304:R473–87. 10.1152/ajpregu.00355.2012. 23389111 PMC4422342

[25] Wagner EM, Sánchez J, McClintock JY, Jenkins J, Moldobaeva A. Inflammation and ischemia-induced lung angiogenesis. Am J Physiol Lung Cell Mol Physiol. 2008; 294:L351–57. 10.1152/ajplung.00369.2007. 18156440

[26] Medford AR, Millar AB. Vascular endothelial growth factor (VEGF) in acute lung injury (ALI) and acute respiratory distress syndrome (ARDS): paradox or paradigm? Thorax. 2006; 61:621–26. 10.1136/thx.2005.040204. 16807391 PMC1828639

[27] Daniel RA, Cardoso VK, Góis E Jr, Parra RS, Garcia SB, Rocha JJ, Féres O. Effect of hyperbaric oxygen therapy on the intestinal ischemia reperfusion injury. Acta Cir Bras. 2011; 26:463–69. 10.1590/s0102-86502011000600010. 22042109

[28] Fu XB, Yang YH, Sun TZ, Gu XM, Jiang LX, Sun XQ, Sheng ZY. Effect of intestinal ischemia-reperfusion on expressions of endogenous basic fibroblast growth factor and transforming growth factor betain lung and its relation with lung repair. World J Gastroenterol. 2000; 6:353–55. 10.3748/wjg.v6.i3.353. 11819596 PMC4688750

[29] Saito A, Horie M, Micke P, Nagase T. The Role of TGF-β Signaling in Lung Cancer Associated with Idiopathic Pulmonary Fibrosis. Int J Mol Sci. 2018; 19:3611. 10.3390/ijms19113611. 30445777 PMC6275044

[30] Hsu YC, Wang LF, Chien YW. Nitric oxide in the pathogenesis of diffuse pulmonary fibrosis. Free Radic Biol Med. 2007; 42:599–607. 10.1016/j.freeradbiomed.2006.11.031. 17291983

[31] Saura M, Zaragoza C, Herranz B, Griera M, Diez-Marqués L, Rodriguez-Puyol D, Rodriguez-Puyol M. Nitric oxide regulates transforming growth factor-beta signaling in endothelial cells. Circ Res. 2005; 97:1115–23. 10.1161/01.RES.0000191538.76771.66. 16239590

[32] Marcos-Ramiro B, García-Weber D, Millán J. TNF-induced endothelial barrier disruption: beyond actin and Rho. Thromb Haemost. 2014; 112:1088–102. 10.1160/TH14-04-0299. 25078148

[33] Genescà M, Sola A, Miquel R, Pi F, Xaus C, Alfaro V, Hotter G. Role of changes in tissular nucleotides on the development of apoptosis during ischemia/reperfusion in rat small bowel. Am J Pathol. 2002; 161:1839–47. 10.1016/S0002-9440(10)64460-4. 12414530 PMC1850803

[34] Sun Y, Gao Q, Wu N, Li SD, Yao JX, Fan WJ. Protective effects of dexmedetomidine on intestinal ischemia-reperfusion injury. Exp Ther Med. 2015; 10:647–52. 10.3892/etm.2015.2561. 26622369 PMC4509460

[35] Albertine KH, Soulier MF, Wang Z, Ishizaka A, Hashimoto S, Zimmerman GA, Matthay MA, Ware LB. Fas and fas ligand are up-regulated in pulmonary edema fluid and lung tissue of patients with acute lung injury and the acute respiratory distress syndrome. Am J Pathol. 2002; 161:1783–96. 10.1016/S0002-9440(10)64455-0. 12414525 PMC1850801

[36] Mura M, Andrade CF, Han B, Seth R, Zhang Y, Bai XH, Waddell TK, Hwang D, Keshavjee S, Liu M. Intestinal ischemia-reperfusion-induced acute lung injury and oncotic cell death in multiple organs. Shock. 2007; 28:227–38. 10.1097/01.shk.0000278497.47041.e3. 17666944

[37] Bala M, Kashuk J, Moore EE, Kluger Y, Biffl W, Gomes CA, Ben-Ishay O, Rubinstein C, Balogh ZJ, Civil I, Coccolini F, Leppaniemi A, Peitzman A, et al. Acute mesenteric ischemia: guidelines of the World Society of Emergency Surgery. World J Emerg Surg. 2017; 12:38. 10.1186/s13017-017-0150-5. 28794797 PMC5545843

[38] Impellizzeri D, Cordaro M, Campolo M, Gugliandolo E, Esposito E, Benedetto F, Cuzzocrea S, Navarra M. Anti-inflammatory and Antioxidant Effects of Flavonoid-Rich Fraction of Bergamot Juice (BJe) in a Mouse Model of Intestinal Ischemia/Reperfusion Injury. Front Pharmacol. 2016; 7:203. 10.3389/fphar.2016.00203. 27471464 PMC4945634

[39] Ito K, Ozasa H, Horikawa S. Edaravone protects against lung injury induced by intestinal ischemia/reperfusion in rat. Free Radic Biol Med. 2005; 38:369–74. 10.1016/j.freeradbiomed.2004.10.029. 15629865

[40] Zhao H, Montalto MC, Pfeiffer KJ, Hao L, Stahl GL. Murine model of gastrointestinal ischemia associated with complement-dependent injury. J Appl Physiol (1985). 2002; 93:338–45. 10.1152/japplphysiol.00159.2002. 12070223

[41] Strand-Amundsen RJ, Reims HM, Reinholt FP, Ruud TE, Yang R, Høgetveit JO, Tønnessen TI. Ischemia/reperfusion injury in porcine intestine - Viability assessment. World J Gastroenterol. 2018; 24:2009–23. 10.3748/wjg.v24.i18.2009. 29760544 PMC5949714

[42] Kim JH, Kim J, Chun J, Lee C, Im JP, Kim JS. Role of iRhom2 in intestinal ischemia-reperfusion-mediated acute lung injury. Sci Rep. 2018; 8:3797. 10.1038/s41598-018-22218-8. 29491382 PMC5830505

[43] Lagan AL, Melley DD, Evans TW, Quinlan GJ. Pathogenesis of the systemic inflammatory syndrome and acute lung injury: role of iron mobilization and decompartmentalization. Am J Physiol Lung Cell Mol Physiol. 2008; 294:L161–74. 10.1152/ajplung.00169.2007. 18055843

[44] Drakopanagiotakis F, Xifteri A, Polychronopoulos V, Bouros D. Apoptosis in lung injury and fibrosis. Eur Respir J. 2008; 32:1631–38. 10.1183/09031936.00176807. 19043009

[45] Patel BV, Wilson MR, Takata M. Resolution of acute lung injury and inflammation: a translational mouse model. Eur Respir J. 2012; 39:1162–70. 10.1183/09031936.00093911. 22005920 PMC3568398

[46] Soares ROS, Losada DM, Jordani MC, Évora P, Castro-E-Silva O. Ischemia/Reperfusion Injury Revisited: An Overview of the Latest Pharmacological Strategies. Int J Mol Sci. 2019; 20:5034. 10.3390/ijms20205034. 31614478 PMC6834141

[47] Egami K, Murohara T, Aoki M, Matsuishi T. Ischemia-induced angiogenesis: role of inflammatory response mediated by P-selectin. J Leukoc Biol. 2006; 79:971–76. 10.1189/jlb.0805448. 16641139

[48] Markel TA, Crafts TD, Jensen AR, Hunsberger EB, Yoder MC. Human mesenchymal stromal cells decrease mortality after intestinal ischemia and reperfusion injury. J Surg Res. 2015; 199:56–66. 10.1016/j.jss.2015.06.060. 26219205

[49] Li L, Liu H, Xu C, Deng M, Song M, Yu X, Xu S, Zhao X. VEGF promotes endothelial progenitor cell differentiation and vascular repair through connexin 43. Stem Cell Res Ther. 2017; 8:237. 10.1186/s13287-017-0684-1. 29065929 PMC5655878

[50] Slevin M, Krupinski J, Slowik A, Rubio F, Szczudlik A, Gaffney J. Activation of MAP kinase (ERK-1/ERK-2), tyrosine kinase and VEGF in the human brain following acute ischaemic stroke. Neuroreport. 2000; 11:2759–64. 10.1097/00001756-200008210-00030. 10976958

[51] Pittet JF, Griffiths MJ, Geiser T, Kaminski N, Dalton SL, Huang X, Brown LA, Gotwals PJ, Koteliansky VE, Matthay MA, Sheppard D. TGF-beta is a critical mediator of acute lung injury. J Clin Invest. 2001; 107:1537–44. 10.1172/JCI11963. 11413161 PMC200192

[52] Saura M, Zaragoza C, Cao W, Bao C, Rodríguez-Puyol M, Rodríguez-Puyol D, Lowenstein CJ. Smad2 mediates transforming growth factor-beta induction of endothelial nitric oxide synthase expression. Circ Res. 2002; 91:806–13. 10.1161/01.res.0000040397.23817.e5. 12411395

[53] Dumont EA, Hofstra L, van Heerde WL, van den Eijnde S, Doevendans PA, DeMuinck E, Daemen MA, Smits JF, Frederik P, Wellens HJ, Daemen MJ, Reutelingsperger CP. Cardiomyocyte death induced by myocardial ischemia and reperfusion: measurement with recombinant human annexin-V in a mouse model. Circulation. 2000; 102:1564–68. 10.1161/01.cir.102.13.1564. 11004148

[54] Dumont EA, Reutelingsperger CP, Smits JF, Daemen MJ, Doevendans PA, Wellens HJ, Hofstra L. Real-time imaging of apoptotic cell-membrane changes at the single-cell level in the beating murine heart. Nat Med. 2001; 7:1352–55. 10.1038/nm1201-1352. 11726977

[55] Eefting F, Rensing B, Wigman J, Pannekoek WJ, Liu WM, Cramer MJ, Lips DJ, Doevendans PA. Role of apoptosis in reperfusion injury. Cardiovasc Res. 2004; 61:414–26. 10.1016/j.cardiores.2003.12.023. 14962473

[56] Liu G, Wang T, Wang T, Song J, Zhou Z. Effects of apoptosis-related proteins caspase-3, Bax and Bcl-2 on cerebral ischemia rats. Biomed Rep. 2013; 1:861–67. 10.3892/br.2013.153. 24649043 PMC3917099

[57] Campolo M, Di Paola R, Impellizzeri D, Crupi R, Morittu VM, Procopio A, Perri E, Britti D, Peli A, Esposito E, Cuzzocrea S. Effects of a polyphenol present in olive oil, oleuropein aglycone, in a murine model of intestinal ischemia/reperfusion injury. J Leukoc Biol. 2013; 93:277–87. 10.1189/jlb.0712317. 23233730

[58] Jalkanen AJ, Puttonen KA, Venäläinen JI, Sinervä V, Mannila A, Ruotsalainen S, Jarho EM, Wallén EA, Männistö PT. Beneficial effect of prolyl oligopeptidase inhibition on spatial memory in young but not in old scopolamine-treated rats. Basic Clin Pharmacol Toxicol. 2007; 100:132–38. 10.1111/j.1742-7843.2006.00021.x. 17244263

[59] Khadaroo RG, Fortis S, Salim SY, Streutker C, Churchill TA, Zhang H. I-FABP as biomarker for the early diagnosis of acute mesenteric ischemia and resultant lung injury. PLoS One. 2014; 9:e115242. 10.1371/journal.pone.0115242. 25541714 PMC4277349

[60] Lai YJ, Chen PR, Huang YL, Hsu HH. Unique wreath-like smooth muscle proliferation of the pulmonary vasculature in pulmonary veno-occlusive disease versus pulmonary arterial hypertension. J Formos Med Assoc. 2020; 119:300–09. 10.1016/j.jfma.2019.05.019. 31202500

[61] Casili G, Lanza M, Filippone A, Campolo M, Paterniti I, Cuzzocrea S, Esposito E. Dimethyl fumarate alleviates the nitroglycerin (NTG)-induced migraine in mice. J Neuroinflammation. 2020; 17:59. 10.1186/s12974-020-01736-1. 32066464 PMC7469611

[62] Campolo M, Filippone A, Biondo C, Mancuso G, Casili G, Lanza M, Cuzzocrea S, Esposito E, Paterniti I. TLR7/8 in the Pathogenesis of Parkinson’s Disease. Int J Mol Sci. 2020; 21:9384. 10.3390/ijms21249384. 33317145 PMC7763162

[63] Campolo M, Siracusa R, Cordaro M, Filippone A, Gugliandolo E, Peritore AF, Impellizzeri D, Crupi R, Paterniti I, Cuzzocrea S. The association of adelmidrol with sodium hyaluronate displays beneficial properties against bladder changes following spinal cord injury in mice. PLoS One. 2019; 14:e0208730. 10.1371/journal.pone.0208730. 30653511 PMC6336272

